# Time-resolved transcriptional profiling of *Trichinella*-infected murine myocytes helps to elucidate host–pathogen interactions in the muscle stage

**DOI:** 10.1186/s13071-021-04624-6

**Published:** 2021-03-01

**Authors:** Xiaoxiang Hu, Xiaolei Liu, Chen Li, Yulu Zhang, Chengyao Li, Yanfeng Li, Yingxi Chen, Heng Guo, Xue Bai, Mingyuan Liu

**Affiliations:** 1 Key Laboratory of Zoonosis Research, Ministry of Education, Institute of Zoonosis, College of Veterinary Medicine, Jilin University, Changchun, 130062 China; 2 Beijing Hi-Tech Institute, Beijing, 100085 China; 3 Jiangsu Co-innovation Center for Prevention and Control of Important Animal Infectious Diseases and Zoonoses, Yangzhou, Jiangsu China

**Keywords:** *Trichinella spiralis*, Transcriptome, RNA sequencing, Host–pathogen interplay

## Abstract

**Background:**

Parasites of the genus *Trichinella* are the pathogenic agents of trichinellosis, which is a widespread and severe foodborne parasitic disease. *Trichinella spiralis* resides primarily in mammalian skeletal muscle cells. After invading the cells of the host organism, *T. spiralis* must elude or invalidate the host’s innate and adaptive immune responses to survive. It is necessary to characterize the pathogenesis of trichinellosis to help to prevent the occurrence and further progression of this disease. The aims of this study were to elucidate the mechanisms of nurse cell formation, pathogenesis and immune evasion of *T. spiralis*, to provide valuable information for further research investigating the basic cell biology of *Trichinella*-infected muscle cells and the interaction between *T. spiralis* and its host.

**Methods:**

We performed transcriptome profiling by RNA sequencing to identify global changes at 1, 3, 7, 10 and 15 days post-infection (dpi) in gene expression in the diaphragm after the parasite entered and persisted within the murine myocytes; the mice were infected by intravenous injection of newborn larvae. Gene expression analysis was based on the alignment results. Differentially expressed genes (DEGs) were identified based on their expression levels in various samples, and functional annotation and enrichment analysis were performed.

**Results:**

The most extensive and dynamic gene expression responses in host diaphragms were observed during early infection (1 dpi). The number of DEGs and genes annotated in the Kyoto Encyclopedia of Genes and Genomes and Gene Ontology databases decreased significantly in the infected mice compared to the uninfected mice at 3 and 7 dpi, suddenly increased sharply at 10 dpi, and then decreased to a lower level at 15 dpi, similar to that observed at 3 and 7 dpi. The massive initial reaction of the murine muscle cells to *Trichinella* infection steadied in the later stages of infection, with little additional changes detected for the remaining duration of the studied process. Although there were hundreds of DEGs at each time point, only 11 genes were consistently up- or downregulated at all 5 time points.

**Conclusions:**

The gene expression patterns identified in this study can be employed to characterize the coordinated response of *T. spiralis*-infected myocytes in a time-resolved manner. This comprehensive dataset presents a distinct and sensitive picture of the interaction between host and parasite during intracellular infection, which can help to elucidate how pathogens evade host defenses and coordinate the biological functions of host cells to survive in the mammalian environment.

**Graphical Abstract:**

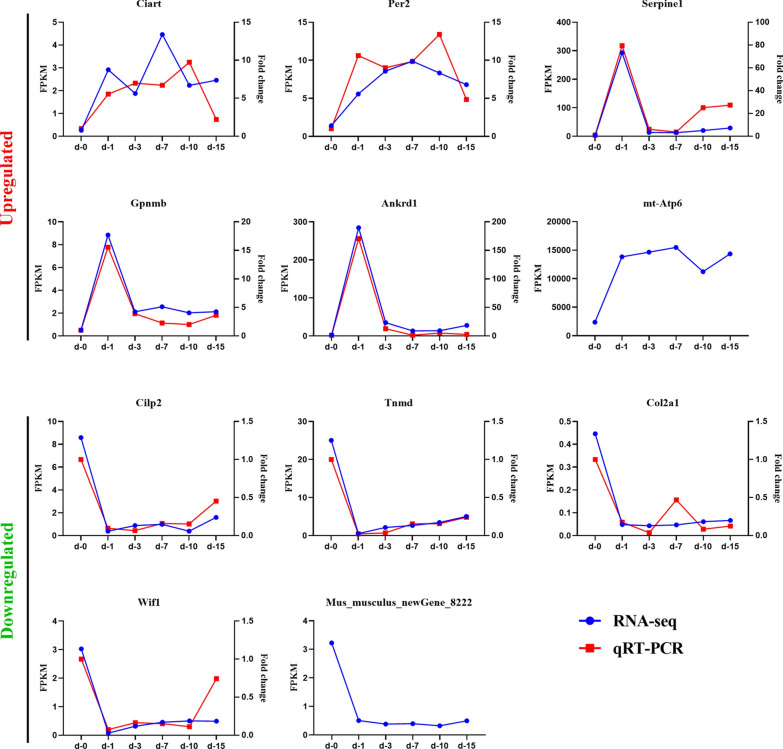

## Background

Trichinellosis is a widespread and severe foodborne parasitic disease caused by the consumption of raw or undercooked pork or its products which contain infective larvae of *Trichinella* nematodes [1]. *Trichinella spiralis*, an intracellular parasitic nematode, is capable of infecting more than 150 species of animals, including humans [2]. The life cycle of *T. spiralis* comprises three stages: newborn larvae (NBL), muscle larvae (ML) and adult worms [3]. After infection, the infective ML molt into adults, mate and reproduce. Next, the cyetic females release NBL, which migrate and invade skeletal myocytes. NBL grow rapidly, cause extensive damage to host myocytes and transform them into a new form, known as nurse cells [4]. Infection with *T. spiralis* can trigger a series of dynamic reactions in the host, resulting in changes in gene expression patterns in muscle, which enable *T. spiralis* to successfully colonize the body. It has been reported that some of the events that occur during nurse cell formation happen in parallel with those occurring during myocyte regeneration/repair [5]. The process of nurse cell formation is accompanied by many biological events in the myocyte, including the activation and proliferation of satellite cells, muscle damage and regeneration, redifferentiation and transformation of the infected muscle cells, and apoptosis leading to changes in the cytoplasm (replacement of basophilic cytoplasm, which originates from the damaged muscle cell, by eosinophilic cytoplasm, which originates from proliferative satellite cells). Infection by *T. spiralis* causes nuclear hypertrophy; these nuclei have been shown to have a mean DNA content of 4 N, which implies long-term arrest of the cycle of the infected cell at G2/M [6]. In addition, the synthesis of type I, IV, and VI collagen, which are crucial components of the nurse cell collagen capsule, significantly increase during *T. spiralis* infection [7].

To search for the candidate genes responsible for the formation of nurse cells, a cDNA microarray analysis was performed on muscle tissues sampled before and after *Trichinella* infection, to show the kinetics of the genes mobilized in response to *Trichinella* infection and provide clues regarding candidate genes in muscle conversion [8]. More recently, researchers analyzed the changes in primary myoblasts induced by ML excretory–secretory products. Microarray analyses disclosed expression variations in muscle cell differentiation, proliferation, cytoskeleton organization, cell motion, transcription, cell cycle, apoptosis and signaling pathways, such as the mitogen-activated protein kinase, Janus kinase/signal transducers and activators of transcription, Wingless/*int1* and phosphatidylinositol 3 kinase/protein kinase B pathways, thereby providing novel information regarding how *T. spiralis* can modify the standard course of skeletal muscle renovation after NBL aggression to achieve nurse cell formation [9].

To analyze the interaction between *T. spiralis* and host myocytes in greater depth at different time points during *T. spiralis* development after invasion, we conducted a comprehensive and in-depth host transcriptome study after transcriptome sequencing of *T. spiralis* by using advanced RNA sequencing (RNA-seq) technology. Unlike the genome, which has a static state and exhibits relative stability, the transcriptome can change with the development of the species itself or with changes in external environmental conditions. The gene expression of the host may vary considerably in different developmental stages or environments. Therefore, the transcriptome has strong temporal and spatial characteristics. Gene transcription levels change as a result of a dynamic relationship between the host genome and external physical characteristics, and can reflect a given developmental or physiological stage. Hence we can obtain a stronger understanding of the characteristics and regulatory mechanisms of a parasite by characterizing the changes in the transcriptome of its host. For example, mouse genome-wide transcriptional responses to infection with two strains of *Plasmodium yoelii* were investigated and deviations in innate response approaches matching strain-specific disease phenotypes were elucidated, demonstrating that a robust type I interferon response contributed to a decrease in parasitemia in N67-infected mice [10]. An increasing number of studies have indicated the value of studying host transcriptome changes to elucidate host-parasite interactions.

A global and kinetic analysis of gene expression profiles of *T. spiralis*-infected muscle with RNA-seq were performed in this study. The time points (1, 3, 7, 10 and 15 days post-intravenous injection of NBL, which are equivalent to about 7, 10, 13, 16 and 21 days post-oral infection) of observation correspond to the duration of nurse cell formation. This study attempted to elucidate the mechanisms of nurse cell formation, pathogenesis and immune evasion, to provide valuable information for further research investigating the basic cell biology of *Trichinella*-infected muscle cells and the interaction between *T. spiralis* and its host. Analyzing differentially expressed genes (DEGs) and pathways may help to establish a foundation for determining the invasion and parasitic mechanisms employed by *T. spiralis*, and may have significance for pathological and immunological research on *T. spiralis* and the screening of therapeutic drugs for trichinellosis.

## Methods

### Mice and *T. spiralis* infection

BALB/c mice (6–8 weeks old) were obtained from the Experimental Animal Center of the College of Basic Medical Sciences, Jilin University (Changchun, China). To obtain more significant transcriptome data, synchronized *T. spiralis* infections were initiated with NBL produced by adult female parasites* in vitro*, as described previously [11]. Mice injected with approximately 40,000 NBL each via tail vein were sacrificed at 1, 3, 7, 10 and 15 dpi. Uninfected mice (0 dpi) and NBL that were not injected into mice were employed as controls. There were three biological replicates for each group at every time point.

### RNA extraction and qualification

Total RNA was extracted from mice diaphragms or NBL using RNAiso Plus (TaKaRa) according to the manufacturer’s recommendations. RNA concentration and purity were determined by NanoDrop 2000 (Thermo Fisher Scientific, Wilmington, DE). RNA integrity was evaluated using the RNA Nano 6000 Assay Kit of the Agilent Bioanalyzer 2100 system (Agilent Technologies, CA, USA).

### Library preparation for transcriptome sequencing

A total of 1 μg RNA per sample was used as input material for the RNA sample preparation. Sequencing libraries were generated using the NEBNext Ultra RNA Library Prep Kit for Illumina (NEB, USA) following the manufacturer’s instructions, and index codes were added to attribute sequences to each sample. Briefly, mRNA was purified from total RNA using poly-T oligo-attached magnetic beads. Fragmentation was conducted using divalent cations under elevated temperature in NEBNext First Strand Synthesis Reaction Buffer (5X). First-strand cDNA was synthesized using random hexamer primers and M-MuLV reverse transcriptase. Second-strand cDNA synthesis was subsequently performed employing DNA polymerase I and RNase H. The remaining overhangs were converted into blunt ends via exonuclease/polymerase activities. After adenylation of the 3′ ends of DNA fragments, NEBNext adaptors with hairpin loop structures were ligated for hybridization preparation. To select cDNA fragments preferentially measuring 240 bp in length, the library fragments were purified with the AMPure XP system (Beckman Coulter, Beverly, MA). Then, 3 μl USER Enzyme (NEB, USA) was utilized with size-selected, adaptor-ligated cDNA at 37 °C for 15 min followed by 5 min at 95 °C before polymerase chain reaction (PCR) analysis. Then, PCR was performed with Phusion High-Fidelity DNA polymerase, universal PCR primers and Index (X) primer. Finally, PCR products were purified (AMPure XP system) and library quality was assessed using the Agilent Bioanalyzer 2100 system.

### Clustering and sequencing

The clustering of the index-coded samples was performed on a cBot Cluster Generation System using TruSeq PE Cluster Kit v4-cBot-HS (Illumina) according to the manufacturer’s instructions. After cluster generation, the library preparations were sequenced on an Illumina platform and paired-end reads were generated.

### Quality control

Raw data (raw reads) in fastq format were first processed through in-house Perl scripts. In this step, clean data (clean reads) were obtained by removing low-quality reads and reads containing the adaptor, poly-N, from raw data. In the meantime, the Q20, Q30, GC content and sequence duplication level of the clean data were calculated. All downstream analyses were based on clean, high-quality data.

### Comparative analysis

Clean reads were subsequently mapped to the reference genome sequence (GRCm38_release95). Only reads with a perfect match or one mismatch were further analyzed and annotated based on the reference genome. Hisat2 tools software was used for mapping with the mouse reference genome.

### Gene functional annotation

Gene function was annotated based on the following databases: National Center for Biotechnology Information [non-redundant protein sequences (Nr); non-redundant nucleotide sequences (Nt)]; Pfam (protein families); Eukaryotic Orthologous Groups/Clusters of Orthologous Groups (KOG/COG); Swiss-Prot (a manually annotated and reviewed protein sequence database); Kyoto Encyclopedia of Genes and Genomes (KEGG) ortholog database (KO); Gene Ontology (GO).

### Quantification of gene expression levels

Fragments per kilobase of transcript per million fragments mapped reads (FPKM) were used to quantify the gene expression levels. The formula is as follows:

$${\text{FPKM = }}\frac{{{\text{cDNA}}\;{\text{Fragments}}}}{{{\text{Mapped}}\,{\text{Fragments }}\left( {\text{Millions}} \right) * {\text{Transcript}}\;{\text{Length}}\;\left( {\text{kb}} \right)}}$$

### Differential expression analysis

Differential expression analysis of the two groups was performed employing edgeR. Statistical routines for determining differential expression in digital gene expression data were provided by edgeR using a model based on the negative binomial distribution. The resulting* p*-values were adjusted by using Benjamini and Hochberg’s approach for controlling the false discovery rate. Genes with adjusted *p*-values < 0.01, as determined by edgeR, were considered to be differentially expressed.

### KEGG pathway enrichment analysis

KEGG is a database that can be used to understand the high-level functions and utilities of biological systems, such as cells, organisms and ecosystems, from molecular-level information, especially large-scale molecular datasets generated by genome sequencing and other high-throughput experimental technologies (http://www.genome.jp/kegg/). We tested the statistical enrichment of DEGs in KEGG pathways employing KEGG Orthology Based Annotation System software.

### GO enrichment analysis

GO enrichment analysis of the DEGs was performed by the GOseq R package-based Wallenius’ noncentral hypergeometric distribution, which can adjust for gene length bias in DEGs.

### Verification of specific transcripts by quantitative real-time PCR

To verify the expression levels of mRNA, reverse transcription was performed using the QuantiNova Reverse Transcription Kit (QIAGEN, Germany) according to the supplier’s recommendations. The resulting cDNA was analyzed by quantitative real-time PCR using an Applied Biosystems StepOnePlus Real-Time PCR System (Thermo Fisher Scientific, USA). The primer sequences utilized in this study are listed in Table 1. Each sample was assayed in triplicate wells on the same plate for all genes. Each reaction contained FastStart Essential DNA Green Master (Roche, Germany), forward primer (0.2 μM), reverse primer (0.2 μM), and cDNA template to a final volume of 20 μl in nuclease-free water. Amplification of all genes was performed under the following conditions: a holding time of 95 °C for 10 min followed by 40 cycles of denaturation at 95 °C for 15 s and primer annealing at 59 °C for 30 s. mRNA fold expression values were analyzed using the relative threshold cycle (Ct) (2^−ΔΔCt^) method [12, 13].

**Table 1:** Primer sequences used for the quantitative real-time polymerase chain reaction

Gene	Primer sequence (5′ → 3′)
*GAPDH*	Forward 5′-TACCCCCAATGTGTCCGTC-3′
	Reverse 5′-AAGAGTGGGAGTTGCTGTTGAAG-3′
*Ciart*	Forward 5′-GTTGCATCCTATGTCCGCCTGTC-3′
	Reverse 5′-TGGCTAGTCATCTGTGGCTCTGG-3′
*Per2*	Forward 5′-GAAACTGAAGTCAAAACGCGTC-3′
	Reverse 5′-GTCCCTGGTGTGGATACTATTC-3′
*Serpine1*	Forward 5′-ATCTTGGATGCTGAACTCATCA-3′
	Reverse 5′-GAGAGAACTTAGGCAGGATGAG-3′
*Gpnmb*	Forward 5′-GATCTCTATCCCTGGCAAAGAC-3′
	Reverse 5′-CAGTTTCCTATTGGCTTGTACG-3′
*Ankrd1*	Forward 5′-GCTGTGAGGCTGAACCGCTATAAG-3′
	Reverse 5′-CCAGCACAGTTCTTGACCTTGAGG-3′
*Cilp2*	Forward 5′-GACAAGTACGAGTACGACGTG-3
	Reverse 5′-TTGTGTGAACGCACCATGTA-3′
*Tnmd*	Forward 5′-GAACAGTCAGTGATTTGGGTTC-3′
	Reverse 5′-GGTCACATTATCGCAAATCTCC-3′
*Col2a1*	Forward 5′-TACTGGAGTGACTGGTCCTAAG-3′
	Reverse 5′-AACACCTTTGGGACCATCTTTT-3′
*Wif1*	Forward 5′-GAAATGGAGGCTTTTGTAACGA-3′
	Reverse 5′-GCAAATACATTTTCCCGGGTAA-3′

*GADPH* Glyceraldehyde-3-phosphate dehydrogenase; *Ciart* circadian-associated repressor of transcription; *Per2* period circadian clock 2; *Serpine1* serine (or cysteine) peptidase inhibitor, clade E, member 1; *Gpnmb* glycoprotein (transmembrane) nmb; *Ankrd1* ankyrin repeat domain 1 (cardiac muscle); *Cilp2* cartilage intermediate layer protein 2; *Tnmd* tenomodulin; *Col2a1* collagen, type II, alpha 1; *Wif1* Wnt inhibitory factor 1

### Statistical analysis

Statistical analysis was performed using GraphPad Prism 8 software for Windows. Comparisons among the treatment and control groups were analyzed using one-way ANOVA. Data are expressed as the mean ± SD of three replicate groups.

## Results

### Infection dynamics and global transcription patterns

Transcriptome profiling using RNA-seq was employed to identify global variations in gene expression over the course of infection of murine diaphragms with *T. spiralis*. Over 1.5 billion sequence reads were assembled across three independent experiments (biological replicates) and labeled batches 1–3. We obtained 230.43 Gb of clean data after mRNA sequencing for 18 samples, with at least 7.33 Gb clean data for each sample, and a quality score of 93.23%, i.e. percentage of bases with a Q score of ≥ 30, where a score of 30 represents an error rate of 1 in 1000 (see Table 2). Next, the clean data were mapped to the reference genome, with the mapping ratio varying from 95.02% to 97.27% (see Table 3).

**Table 2:** Sequencing data statistics

Samples	Clean reads^a^	Clean bases^b^	GC content^c^ (%)	% ≥ Q30^d^
d-0-1	29,306,627	8,723,905,770	49.41	93.33
d-0-2	29,701,695	8,856,713,318	49.12	93.23
d-0-3	24,573,915	7,325,026,546	49.13	93.51
d-1-1	42,774,938	12,743,577,440	49.50	95.06
d-1-2	47,356,896	14,119,786,038	49.09	94.64
d-1-3	47,632,923	14,229,261,830	49.12	94.83
d-3-1	41,692,065	12,459,755,692	49.22	94.96
d-3-2	44,962,040	13,435,712,256	49.19	95.19
d-3-3	41,808,804	12,496,583,212	49.03	95.04
d-7-1	52,403,887	15,659,838,448	48.70	94.91
d-7-2	44,892,431	13,419,760,674	49.43	94.82
d-7-3	43,566,080	13,011,606,674	48.79	94.77
d-10-1	46,072,970	13,783,739,164	49.41	94.85
d-10-2	53,341,148	15,929,948,426	49.46	94.83
d-10-3	50,069,261	14,950,813,380	49.36	94.67
d-15-1	47,120,191	14,081,050,788	49.71	94.60
d-15-2	41,755,099	12,464,431,734	48.94	94.73
d-15-3	42,645,130	12,738,561,562	49.23	94.92

^a^Paired-end reads of clean data

^b^Total number of bases of clean data

^c^GC content percentage of clean data, i.e. percentage of clean data for bases G and C

^d^Percentage of bases with a Q-score of ≥ 30

**Table 3:** Alignment results of each sample

Samples	Total reads^a^	Mapped reads^b^	Uniq. mapped reads^c^	Multiple map. reads^d^	Reads map to ‘+’^e^	Reads map to ‘–’^f^
d-0-1	58,613,254	56,475,681 (96.35%)	50,859,528 (86.77%)	5,616,153 (9.58%)	25,981,307 (44.33%)	26,076,507 (44.49%)
d-0-2	59,403,390	57,299,237 (96.46%)	51,049,269 (85.94%)	6,249,968 (10.52%)	26,071,598 (43.89%)	26,139,723 (44.00%)
d-0-3	49,147,830	47,414,092 (96.47%)	42,500,765 (86.48%)	4,913,327 (10.00%)	21,732,380 (44.22%)	21,786,530 (44.33%)
d-1-1	85,549,876	82,325,825 (96.23%)	74,360,476 (86.92%)	7,965,349 (9.31%)	38,336,236 (44.81%)	38,490,739 (44.99%)
d-1-2	94,713,792	91,639,498 (96.75%)	81,994,693 (86.57%)	9,644,805 (10.18%)	42,254,746 (44.61%)	42,390,965 (44.76%)
d-1-3	95,265,846	92,379,516 (96.97%)	81,663,964 (85.72%)	10,715,552 (11.25%)	42,047,639 (44.14%)	42,154,318 (44.25%)
d-3-1	83,384,130	80,785,406 (96.88%)	71,651,204 (85.93%)	9,134,202 (10.95%)	36,970,139 (44.34%)	37,058,529 (44.44%)
d-3-2	89,924,080	87,153,989 (96.92%)	77,696,003 (86.40%)	9,457,986 (10.52%)	39,952,614 (44.43%)	40,064,092 (44.55%)
d-3-3	83,617,608	81,334,162 (97.27%)	71,806,277 (85.87%)	9,527,885 (11.39%)	36,949,431 (44.19%)	37,014,840 (44.27%)
d-7-1	104,807,774	101,706,283 (97.04%)	89,439,347 (85.34%)	12,266,936 (11.70%)	46,119,278 (44.00%)	46,223,712 (44.10%)
d-7-2	89,784,862	86,469,953 (96.31%)	78,051,494 (86.93%)	8,418,459 (9.38%)	40,087,913 (44.65%)	40,143,735 (44.71%)
d-7-3	87,132,160	84,256,540 (96.70%)	74,469,393 (85.47%)	9,787,147 (11.23%)	38,286,864 (43.94%)	38,382,981 (44.05%)
d-10-1	92,145,940	89,221,639 (96.83%)	81,900,144 (88.88%)	7,321,495 (7.95%)	42,120,068 (45.71%)	42,160,622 (45.75%)
d-10-2	106,682,296	102,682,416 (96.25%)	92,480,400 (86.69%)	10,202,016 (9.56%)	47,660,016 (44.67%)	47,779,175 (44.79%)
d-10-3	100,138,522	96,653,596 (96.52%)	87,175,782 (87.06%)	9,477,814 (9.46%)	44,725,983 (44.66%)	44,860,821 (44.80%)
d-15-1	94,240,382	90,680,881 (96.22%)	80,900,655 (85.84%)	9,780,226 (10.38%)	41,755,839 (44.31%)	41,819,200 (44.38%)
d-15-2	83,510,198	79,354,215 (95.02%)	70,468,299 (84.38%)	8,885,916 (10.64%)	36,144,364 (43.28%)	36,222,735 (43.38%)
d-15-3	85,290,260	81,572,791 (95.64%)	73,292,073 (85.93%)	8,280,718 (9.71%)	37,623,305 (44.11%)	37,700,361 (44.20%)

^a^Number of reads of clean data, not paired-end reads

^b^Number of reads mapped to the reference genome and the percentage in clean reads

^c^Number of reads mapped uniquely (*Uniq*.) to the reference genome and the percentage in clean reads

^d^Number of reads multiply mapped (map.) to the reference genome and the percentage in clean reads

^e^Number of reads mapped to the sense chain and the percentage in clean reads

^f^Number of reads mapped to the anti-sense chain and the percentage in clean reads

**Fig. 1:**
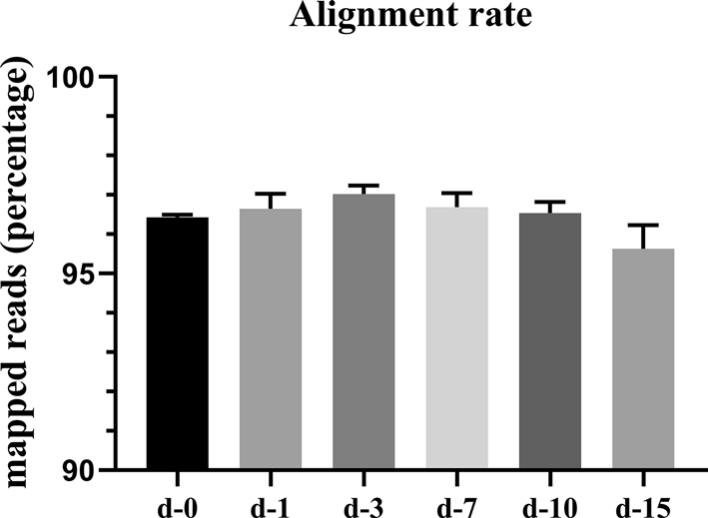
Results of the sequence alignment of the sample sequencing data with the selected reference genome. Diaphragms were collected at 1 (*d–1*), 3 (*d–3*), 7 (*d–7*), 10 (*d–10*), 15 (*d–15*) days post-infection (dpi); uninfected diaphragms were also collected (*d–0*). There was no statistical difference in the percentage of mapped reads at different time points between the uninfected and infected diaphragms

Seeing that there is tiny sequence preservation between mouse and *T. spiralis* and a notably low percentage of *T. spiralis* mapped reads in the clean reads (the highest is merely 1.25%; unpublished data), we were capable of explicitly mapping reads from mouse RNAs from the compound samples to its genome. For each time point, the percentage of reads mapping to the host changed only slightly over the course of the infection. Although there was no significant difference, there was a slight upward and then downward trend in the percentage of murine mapped reads as the infection progressed (Fig. 1). This tiny nonuniformity was observed for the percentage of reads mapping to mouse in all samples, which may help to characterize the variable transcriptional activity of the host. A possible explanation for this phenomenon is that mouse diaphragm cells were more transcriptionally active when first stimulated by the parasite and became less transcriptionally active as the infection progressed.

### Statistical assessment of biological replicates

**Fig. 2:**
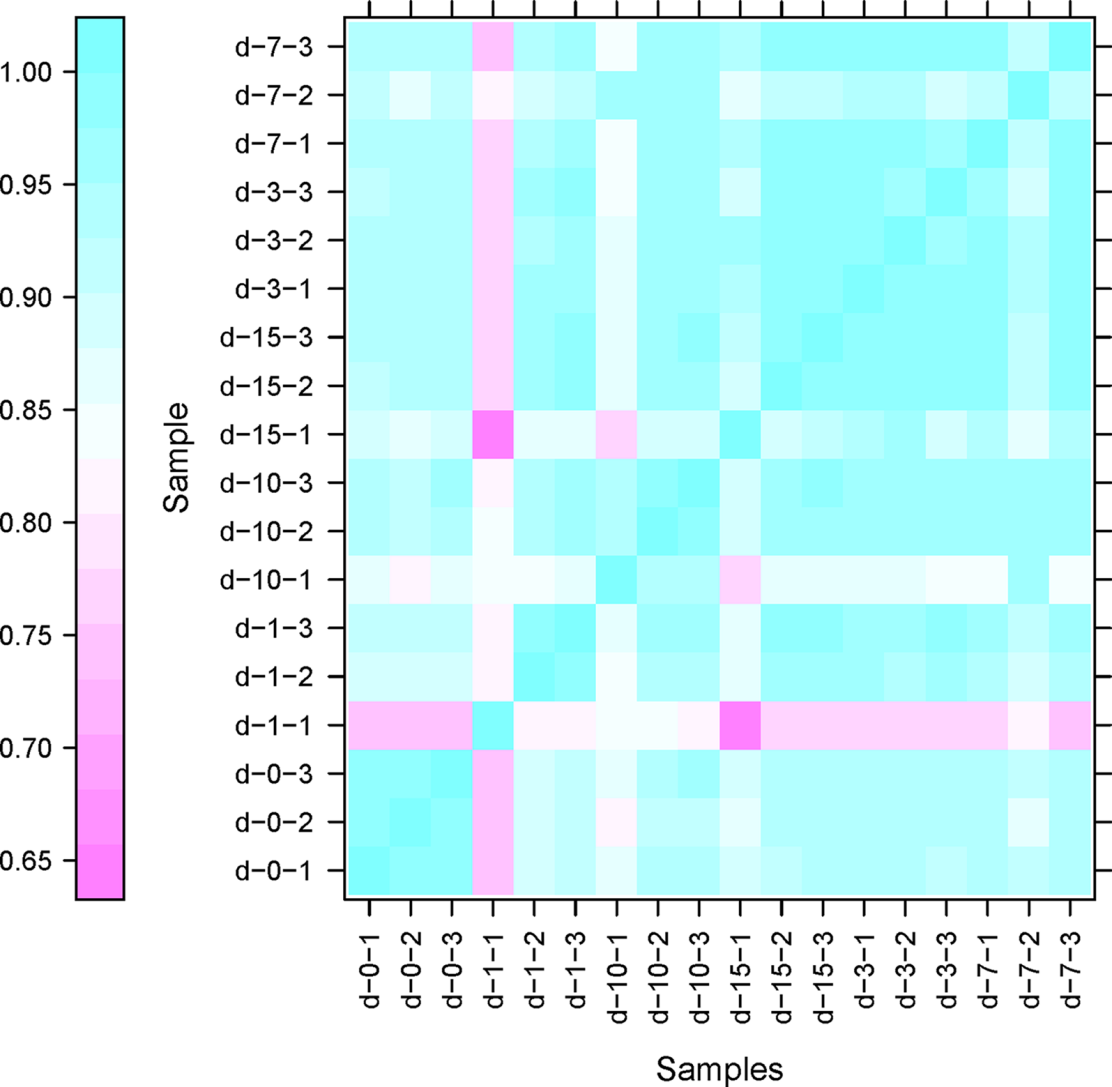
Global gene expression profiles of murine diaphragm host cells. Pearson’s correlation coefficient (*R*^2^) was used as the standard evaluation index of correlation between the biological replicates (replicate number indicated by the* final digit* of the samples). The closer to 1 the *R*^2^, the more similar the two replicates. For other abbreviations, see Fig. 1

Pearson’s correlation coefficient (*R*^2^) is a commonly used evaluation index of correlation between biological replicates [14]. The closer the *R*^2^ is to 1, the more similar the two replicates. We employed a correlation heatmap to visualize the relationship between the experimental datasets (Fig. 2), and found that *R*^2^ between most biological replications was approximately 0.9. This analysis demonstrated that there was a high level of repeatability between the biological replicates exposed to the same experimental conditions (dpi).

### Identification of DEGs in diaphragms

**Fig. 3:**
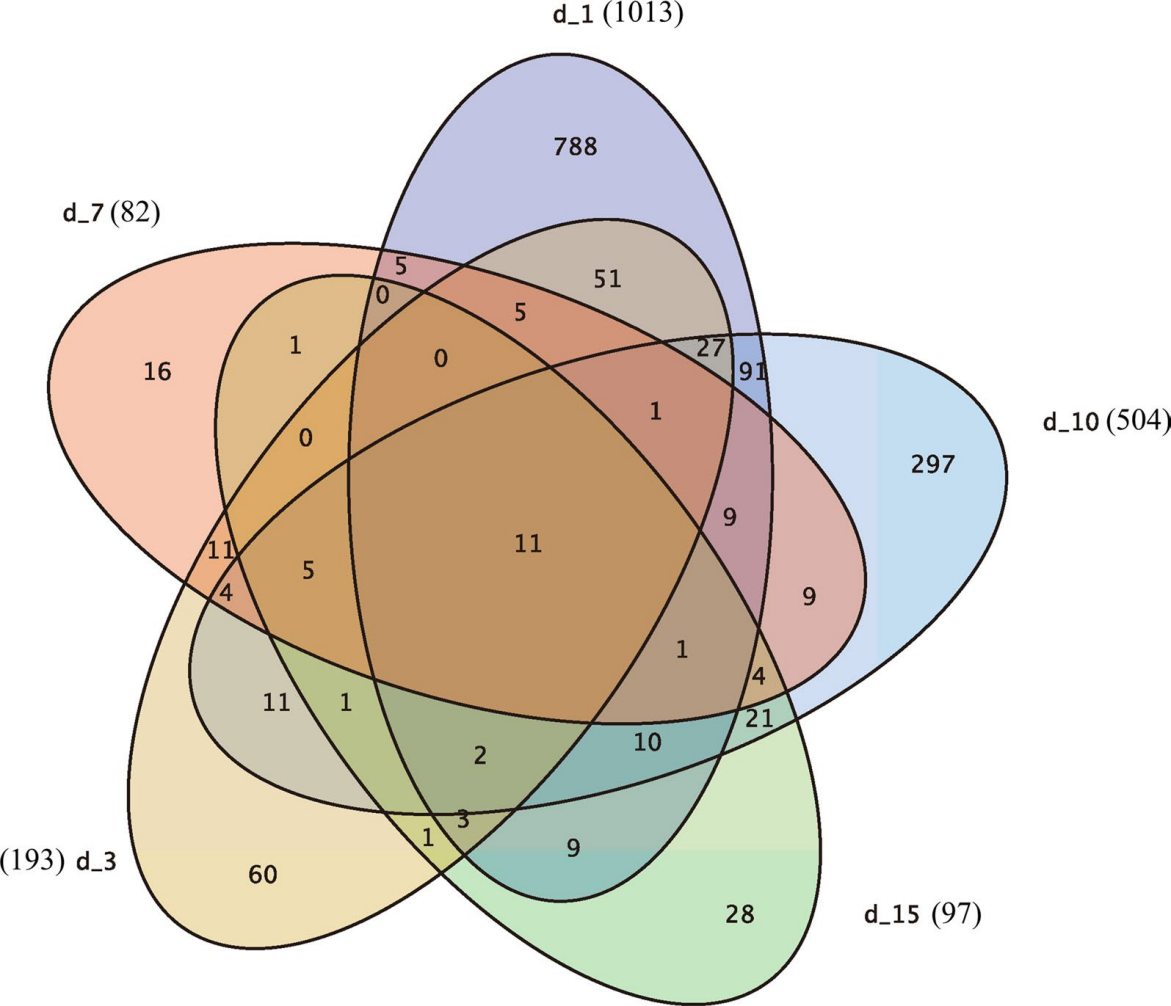
Differentially expressed genes (DEGs) in murine diaphragm cells. The DEG (fold change ≥ 2 and false discovery rate < 0.01) of uninfected* vs* infected mouse diaphragm cells at each time point were compared, and the results displayed as a Venn chart. The complete lists of DEGs are provided in Additional file 1: Table S1. For other abbreviations, see Fig. 1

**Fig. 4:**
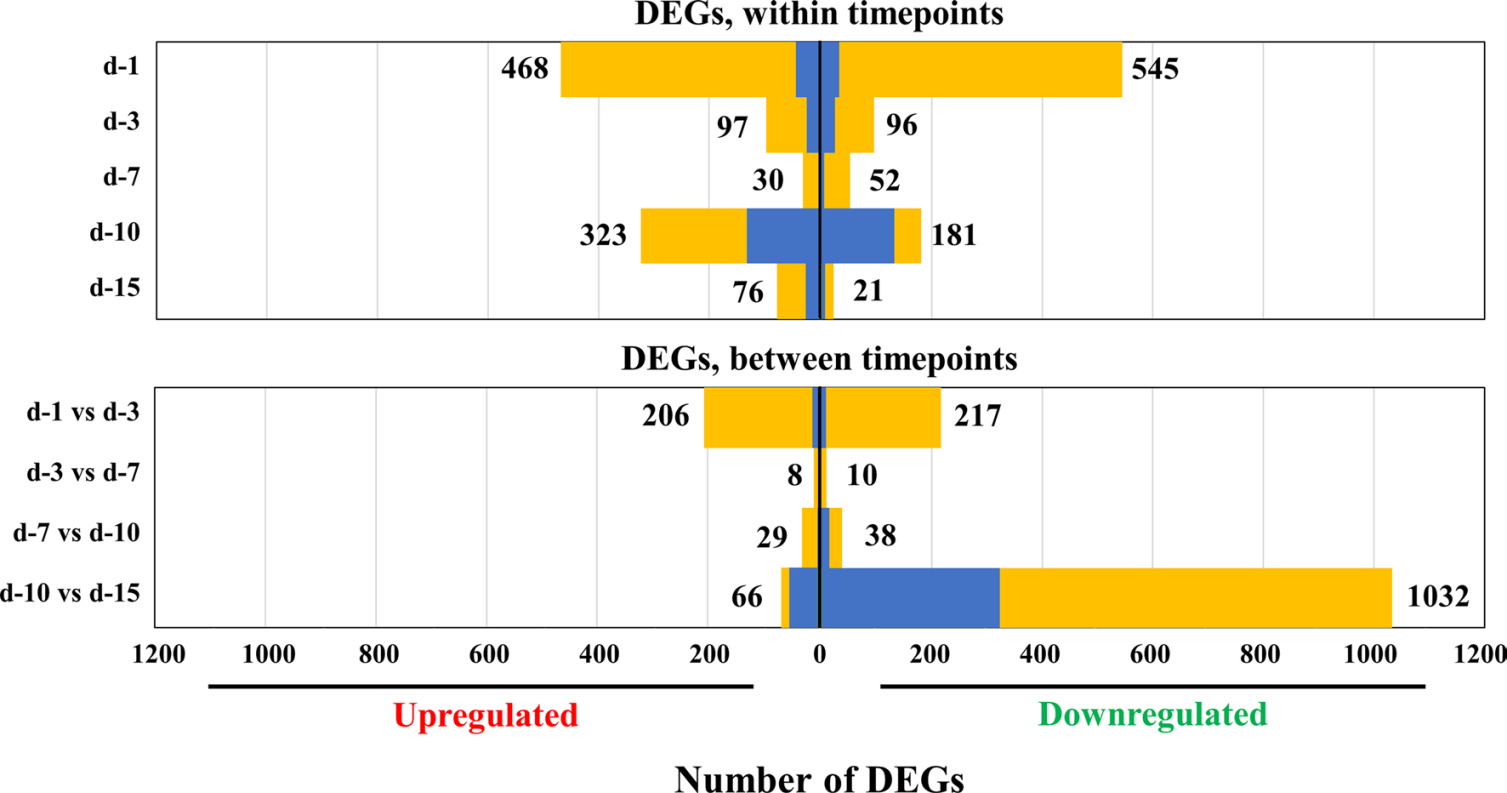
DEGs in murine diaphragm cells. Pairwise comparisons were made to compare DEGs in uninfected* vs* infected mouse samples at each time point (*top*) and between time points (*bottom*). *Box length* indicates the number of upregulated (*left*) or downregulated (*right*) DEGs, with the total number of up- and downregulated genes shown. Genes with > fourfold differential expression (*blue*) or twofold differential expression (*yellow*). For abbreviations, see Figs. 1 and  3

While the heatmap analyses gave an advanced summary of the behavior of the transcriptomes of the murine diaphragms during infection, further investigation was required to assess alterations in the expression levels of individual genes. A differential expression analysis was performed to closely examine the response of the murine diaphragms to *T. spiralis* infection at 0, 1, 3, 7, 10 and 15 dpi. Pairwise comparison within and across individual time points were carried out to compare infected with uninfected diaphragms (Figs. 3, 4).

The most extensive response to infection was acquired at 1 dpi, with 1013 DEGs between the uninfected and infected diaphragms. There was a lesser response at later time points, as reflected by fewer DEGs: 193, 82, 504 and 97 genes at 3, 7, 10 and 15 dpi, respectively. The analysis of up- and downregulated genes for each time point comparison is depicted in Fig. 4 (top panel). Complete DEG lists for each time point are provided in Additional file 1: Table S1. Other than a slight upward and then downward trend in the percentage of mapped reads as the infection progressed as previously mentioned, the number of DEGs in the infected compared to the uninfected diaphragms decreased significantly at 3 and 7 dpi, suddenly increased sharply at 10 dpi, then dropped to a lower level at 15 dpi, which was similar to that observed at 3 and 7 dpi.

An immediate comparison using the superposition of DEGs at each time point demonstrated that the diaphragm response to infection at 1 dpi was notably different to that at the subsequent time points (Fig. 3). Of the 1013 DEGs at 1 dpi, 77.8% (788 genes) were unique. In contrast, 31.1% of genes at 3 dpi (60), 19.5% of genes at 7 dpi [16], 58.9% of genes at 10 dpi (297) and 28.9% of genes at 15 dpi [28] were differentially expressed only at that specific time point. Higher percentages of unique genes were still observed at 1 and 10 dpi. Indeed, only 11 genes were uniformly up- or downregulated at all 5 time points (6 genes upregulated at all time points and 5 genes downregulated at all time points; see Additional file 2: Table S2).

Differential expression analysis across time points was conducted to explore statistical differences in gene expression over time. Comparisons between subsequent time points demonstrated substantial numbers of DEGs from 1 to 3 dpi (423 genes) and between 10 and 15 dpi (1098 genes), but notably few significantly differentially expressed genes were observed between 3 and 7 dpi (18 genes) or between 7 and 10 dpi (67 genes) (Fig. 4, bottom panel). This result indicates that the massive initial reaction of the murine muscle cells to *Trichinella* infection steadied in the later stages of the infection, with few additional changes detected for the period studied, as the infected cells generally maintained their expression pattern. In the late stage of infection, diaphragm DEGs varied dramatically, roughly consistent with the changes of DEGs at each previous time point. These results imply that the response of muscle after NBL entry is not a continuous and steady process with a slow, or drastic, trend, but a process that can show both dramatic changes and then more stable ones, exhibiting a specific rhythm. Except for the rapid changes at the beginning of the infection with the entry of NBL, the response of the host tends to be steady, with sharper changes seen once again at the late stage of infection.

### Eleven genes consistently up- or downregulated at all 5 time points

**Fig. 5:**
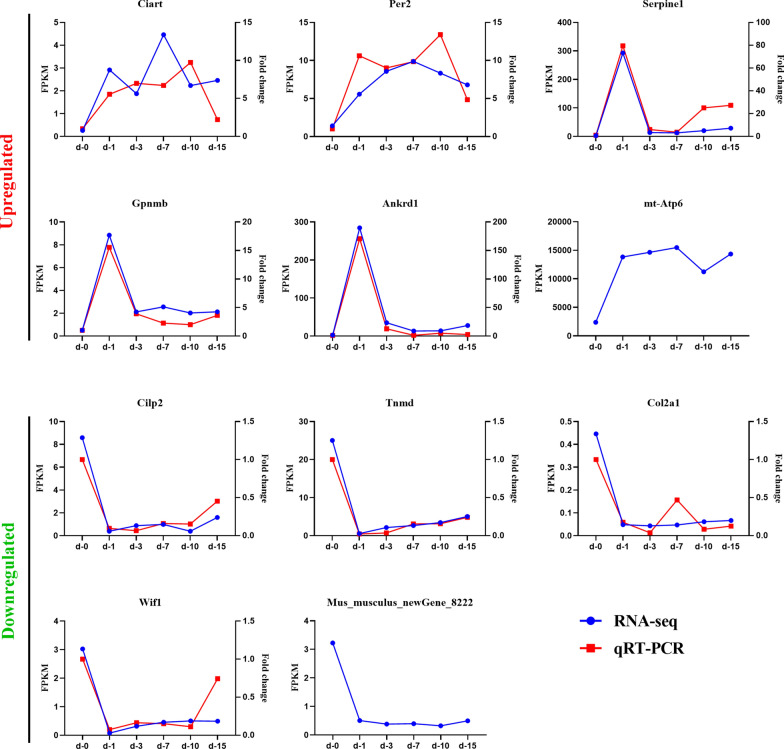
The 11 genes consistently up- or downregulated at all 5 time points. Six genes were upregulated at all time points and 5 genes were downregulated at all time points. Quantification of gene expression levels was evaluated by fragments per kilobase of transcript per million fragments mapped reads (*blue*) and quantitative real-time polymerase chain reaction (*qRT-PCR*) (*red*). The expression of circadian-associated repressor of transcription (*Ciart*); period circadian clock 2 (*Per2*); cartilage intermediate layer protein 2 (*Cilp2*); tenomodulin (*Tnmd*); collagen, type II, alpha 1 (*Col2a1*); and Wnt inhibitory factor 1 (*Wif1*) mRNA in diaphragms of *T. spiralis*-infected mice on 1, 3, 7, 10 and 15 dpi were all significantly different from those of uninfected mice (*P *< 0.0001). For other abbreviations, see Figs. 1 and  3

The 11 genes that were always either upregulated or downregulated at all 5 time points were: circadian-associated repressor of transcription (*Ciart*); period circadian clock 2 (*Per2*); serine (or cysteine) peptidase inhibitor, clade E, member 1 (*Serpine1*); glycoprotein (transmembrane) nmb (*Gpnmb*); ankyrin repeat domain 1 (cardiac muscle) (*Ankrd1*); ATP synthase 6, mitochondrial (*Mtatp6*); cartilage intermediate layer protein 2 (*Cilp2*); tenomodulin (*Tnmd*); collagen, type II, alpha 1 (*Col2a1*); Wnt inhibitory factor 1 (*Wif1*); and* Mus_musculus_newGene_8222*, which is predicted to be ribosomal protein L10 (*Rpl10*). In addition, in spite of the fact that 11 genes were consistently up- or downregulated at all 5 time points, the line graph reflects the disparate variation trend of the expression levels of these genes at each time point (Fig. 5). Among the 6 upregulated genes, expression levels of* Ciart*,* Per2*, and* Mtatp6* increased steadily then subsequently decreased slightly at 10 dpi, while expression levels of the other 3 genes increased sharply on the first day and leveled off at later time points. The other five downregulated genes showed a similar trend, decreasing rapidly at 1 dpi and increasing slowly thereafter without exception.

To verify the genes that were differentially expressed at all 5 time points, the expression levels of 9 genes—*Ciart*,* Per2*,* Serpine1*,* Gpnmb*,* Ankrd1*,* Cilp2*,* Tnmd*,* Col2a1*, and* Wif1*—were analyzed by quantitative real-time PCR. The expression of* Ciart*,* Per2*,* Cilp2*,* Tnmd*,* Col2a1*, and* Wif1* at each time point post-infection was significantly different from that observed in uninfected diaphragms (*P *< 0.0001). The gene expression trends of the 9 genes were generally consistent with the sequencing results, confirming the data obtained in the sequencing analysis (Fig. 5).

### KEGG annotation and pathway enrichment analysis of DEGs

The enriched KEGG pathways were employed to analyze the DEG lists to acquire insights into the host response to infection and how it differs between early and later stages of infection. The cellular processes most influenced at each time point were characterized by KEGG pathway enrichment analysis. The KEGG database was employed for functional annotation of DEGs, and the number of genes annotated in the database for each DEG set was 600 (1 dpi), 126 (3 dpi), 53 (7 dpi), 317 (10 dpi) and 73 (15 dpi). Genes that were differentially expressed > twofold were utilized as input with upregulated and downregulated genes considered respectively. The results of this analysis (*q*-value < 0.01) are reported in Table 4. Early on in the infection (1 dpi), numerous KEGG pathways that were most extremely triggered in infected diaphragms were connected with immune responses, specifically cytokine–cytokine receptor interactions and many signaling pathways, including the tumor necrosis factor, nuclear factor-kappa B, nodulation (NOD)-like receptor, mitogen-activated protein kinase (MAPK), Jak-STAT, HIF-1 and adipocytokine ones. KEGG pathways connected with transcriptional misregulation in cancer and microRNAs in cancer are also present in the list of upregulated genes, indicating that the role of *Trichinella* in cancer warrants further investigation. KEGG pathways downregulated at 1 dpi are mainly related to autoimmune disease, including inflammatory bowel disease (IBD), asthma, rheumatoid arthritis and type I diabetes mellitus. A reduction in the receptors and signaling factors involved in the intestinal immune network for immunoglobulin A production were also observed at 1 dpi. Beyond expectation, the results also showed that the invasion of the NBL into muscle cells may be lead to the suppression of intestinal immunity through the whole body mediation network, and in turn promote the survival of adults in the guts. Other pathways that are downregulated include protein digestion and absorption, antigen processing and presentation, cell adhesion molecule and hematopoietic cell lineage pathways.

**Table 4:** Kyoto Encyclopedia of Genes and Genomes (KEGG) pathway enrichment analysis of murine diaphragms at 5 time points

ID	Description	Gene ratio (%)	Bg ratio (%)	Enrich. factor^a^	*q*-value^b^
d-1, upregulated					
ko04668	TNF signaling pathway	11.56	1.37	8.44	2.06E−11
ko04060	Cytokine-cytokine receptor interaction	10.98	3.19	3.45	0.000183
ko04064	NF-kappa B signaling pathway	7.51	1.59	4.72	0.000183
ko05144	Malaria	4.62	0.66	7.01	0.000626
ko05202	Transcriptional misregulation in cancer	8.67	2.51	3.45	0.000914
ko04621	NOD-like receptor signaling pathway	4.62	0.76	6.09	0.001182
ko05132	Salmonella infection	5.20	1	5.23	0.001182
ko05134	Legionellosis	4.62	0.83	5.55	0.001781
ko04640	Hematopoietic cell lineage	5.78	1.37	4.22	0.002204
ko05206	MicroRNAs in cancer	6.36	1.79	3.55	0.004319
ko04933	AGE-RAGE signaling pathway in diabetic complications	5.20	1.27	4.1	0.004957
ko04010	MAPK signaling pathway	8.67	3.27	2.65	0.007215
ko04380	Osteoclast differentiation	5.78	1.67	3.47	0.007614
ko04630	Jak-STAT signaling pathway	6.36	2.03	3.13	0.008884
ko04066	HIF-1 signaling pathway	5.20	1.44	3.6	0.009357
ko04920	Adipocytokine signaling pathway	4.05	0.91	4.45	0.009608
d-1, downregulated					
ko04974	Protein digestion and absorption	7.14	1.12	6.38	5.46E−06
ko05150	*Staphylococcus aureus* infection	6.63	1.06	6.27	1.07E−05
ko04612	Antigen processing and presentation	6.12	1.18	5.18	0.000166
ko05321	Inflammatory bowel disease	5.10	0.83	6.12	0.000166
ko05145	Toxoplasmosis	6.63	1.47	4.52	0.000166
ko04512	ECM-receptor interaction	5.61	1.05	5.37	0.000166
ko05310	Asthma	4.08	0.68	5.96	0.00131
ko04672	Intestinal immune network for IgA production	4.59	0.90	5.12	0.001329
ko04514	Cell adhesion molecules	7.14	2.13	3.36	0.001439
ko05323	Rheumatoid arthritis	5.61	1.43	3.92	0.00198
ko05152	Tuberculosis	7.65	2.53	3.03	0.002057
ko05332	Graft-versus-host disease	4.08	0.78	5.21	0.002057
ko04640	Hematopoietic cell lineage	5.10	1.37	3.73	0.004764
ko04940	Type I diabetes mellitus	4.08	0.91	4.49	0.004971
d-3, upregulated					
ko04713	Circadian entrainment	13.89	1.19	11.62	0.004997
d-3, downregulated					
ko04974	Protein digestion and absorption	18.42	1.12	16.45	1.37E−05
ko05144	Malaria	10.53	0.66	15.96	0.003842
ko05143	African trypanosomiasis	10.53	0.71	14.84	0.003842
d-7, downregulated					
ko04512	ECM-receptor interaction	23.53	1.05	22.51	0.000396
ko04974	Protein digestion and absorption	23.53	1.12	21.01	0.000396
ko04510	Focal adhesion	23.53	2.60	9.05	0.00683
ko04710	Circadian rhythm	11.76	0.35	33.76	0.009739
d-10, downregulated					
ko04974	Protein digestion and absorption	12.90	1.12	11.52	4.08E−05
ko04512	ECM-receptor interaction	11.29	1.05	10.8	0.000179
ko04932	Non-alcoholic fatty liver disease	12.90	1.94	6.65	0.000855
ko05010	Alzheimer’s disease	12.90	2.24	5.76	0.001788
ko00190	Oxidative phosphorylation	11.29	1.77	6.39	0.001807
ko04151	PI3K-Akt signaling pathway	17.74	4.62	3.84	0.001807
ko05012	Parkinson’s disease	11.29	1.84	6.13	0.001807
ko05016	Huntington’s disease	12.90	2.48	5.21	0.001807

KEGG pathway analysis identified signaling pathways over-represented in *T. spiralis*-infected mouse diaphragms (*q*-value < 0.01) compared to uninfected controls. Genes that were differentially expressed more than twofold were utilized as input with up- and downregulated genes considered respectively. For each enriched KEGG pathway, the ratio of DEGs in the pathway/all DEGs in all pathways (*Gene ratio*) and the ratio of genes in the pathway/all genes in all pathways (*Bg ratio*) are reported

*TNF* Tumor necrosis factor,* ECM* extracellular matrix protein,*NF* nuclear factor,* NOD* nodulation,* AGE–RAGE* advanced glycation endproducts–receptor for advanced glycation endproducts,* JAK–STAT* Janus kinase-signal transducer and activator of transcription pathway,* IgA* immunoglobulin A,* HIF-1* hypoxia-inducible factor 1,* PI3K-Akt* phosphatidylinositol 3-kinase/protein kinase B

^a^Enrichment (*Enrich*.) factors show the proportion of gene ratio to Bg ratio. The bigger the enrichment factor, the more significant the pathway

^b^*q*-value is the* p*-value adjusted by multiple hypothesis testing. The smaller the* q*-value, the more significant the pathway. The DEGs corresponding to the respective enriched KEGG pathway are shown in Additional file 3: Table S3

The enriched KEGG pathways detected at 3, 7, 10 and 15 dpi yielded diverse enrichment patterns compared to those obtained at 1 dpi. There were more pathways downregulated than upregulated at every later time point, but no particularly distinct picture was apparent, except for the ECM-receptor interaction, which was nearly always present. Pathways at these four time points were all downregulated, apart from circadian entrainment at 3 dpi. Furthermore, the number of enriched KEGG pathways at other time points was significantly lower than that at 1 dpi. Similar to the pattern of variation of DEGs, other than the considerable changes at the beginning of the entry of NBL into the host diaphragm, pathway-based enrichment analyses also showed that the muscles were relatively stable before signs of further changes were apparent at the late stage of infection.

A large number of DEGs (1098 genes) were apparent during the transition from 10 to 15 dpi; 1032 of these genes were downregulated, and accounted for 94% of the total DEGs at 10 and 15 dpi. We attempted to determine the cause of this massive and extraordinary change. Specifically, we conducted KEGG enrichment analysis of genes downregulated between 10 and 15 dpi (Table 5), and found that almost all the KEGG pathways during the 10–15 dpi transition were related to amino acid metabolism, although no significant increase in these pathways was observed at the previous time points. This findings indicated that a there were fewer DEGs at 15 dpi because *T. spiralis* had completely developed at this point and stopped absorbing large amounts of energy from the host cells.

**Table 5:** KEGG pathways enriched in murine diaphragms between 10 and 15 dpi

ID	Description	Gene ratio (%)	Bg ratio (%)	Enrich. factor	*q*-value
ko00040	Pentose and glucuronate interconversions	3.75	0.51	7.35	0
ko00260	Glycine, serine and threonine metabolism	4.13	0.57	7.21	0
ko00983	Drug metabolism-other enzymes	4.69	0.73	6.38	0
ko04610	Complement and coagulation cascades	9.19	1.14	8.03	1.36E−31
ko00140	Steroid hormone biosynthesis	8.63	1.12	7.71	1.68E−28
ko00053	Ascorbate and aldarate metabolism	3.75	0.39	9.73	2.12E−14
ko00380	Tryptophan metabolism	3.94	0.57	6.88	1.46E−11
ko00830	Retinol metabolism	9.01	1.19	7.54	3.64E−11
ko05204	Chemical carcinogenesis	8.44	1.27	6.65	4.65E−11
ko00120	Primary bile acid biosynthesis	2.45	0.20	12.25	5.01E−11
ko00982	Drug metabolism-cytochrome P450	6.00	0.93	6.43	9.06E−11
ko00980	Metabolism of xenobiotics by cytochrome P450	5.63	0.93	6.03	9.06E−11
ko03320	PPAR signaling pathway	5.44	1.13	4.804136	1.43E−10
ko04146	Peroxisome	5.07	1.06	4.788544	6.56E−10
ko00071	Fatty acid degradation	3.75	0.66	5.688697	1.26E−08
ko00860	Porphyrin and chlorophyll metabolism	3.19	0.55	5.82445	2.38E−07
ko00591	Linoleic acid metabolism	3.38	0.65	5.22	6.13E−07
ko00590	Arachidonic acid metabolism	4.50	1.16	3.89	1.17E−06
ko00270	Cysteine and methionine metabolism	3.19	0.63	5.03	3.31E−06
ko00500	Starch and sucrose metabolism	3.38	0.76	4.45	1.07E−05
ko04976	Bile secretion	3.56	0.88	4.03	2.58E−05
ko01040	Biosynthesis of unsaturated fatty acids	2.25	0.36	6.24	3.02E−05
ko04950	Maturity onset diabetes of the young	2.06	0.32	6.38	7.86E−05
ko01230	Biosynthesis of amino acids	3.94	1.22	3.23	0.000322
ko00630	Glyoxylate and dicarboxylate metabolism	2.06	0.37	5.53	0.000435
ko00340	Histidine metabolism	1.88	0.32	5.80	0.000786
ko00220	Arginine biosynthesis	1.69	0.26	6.46	0.000854
ko00770	Pantothenate and CoA biosynthesis	1.50	0.22	6.70	0.002209
ko00350	Tyrosine metabolism	2.25	0.52	4.31	0.002898
ko00410	beta-Alanine metabolism	1.88	0.40	4.71	0.006635

KEGG pathway analysis identified pathways over-represented in *T. spiralis*-infected mouse diaphragms (*q*-value < 0.01) between 10 and 15 dpi. Downregulated genes that were differentially expressed more than twofold were utilized as input. The DEGs corresponding to every enriched KEGG pathway are shown in Additional file 4: Table S4

*PPAR* Peroxisome proliferator-activated receptor,* CoA* coenzyme A; for other abbreviations, see Table 4

### GO classification of DEGs

**Fig. 6:**
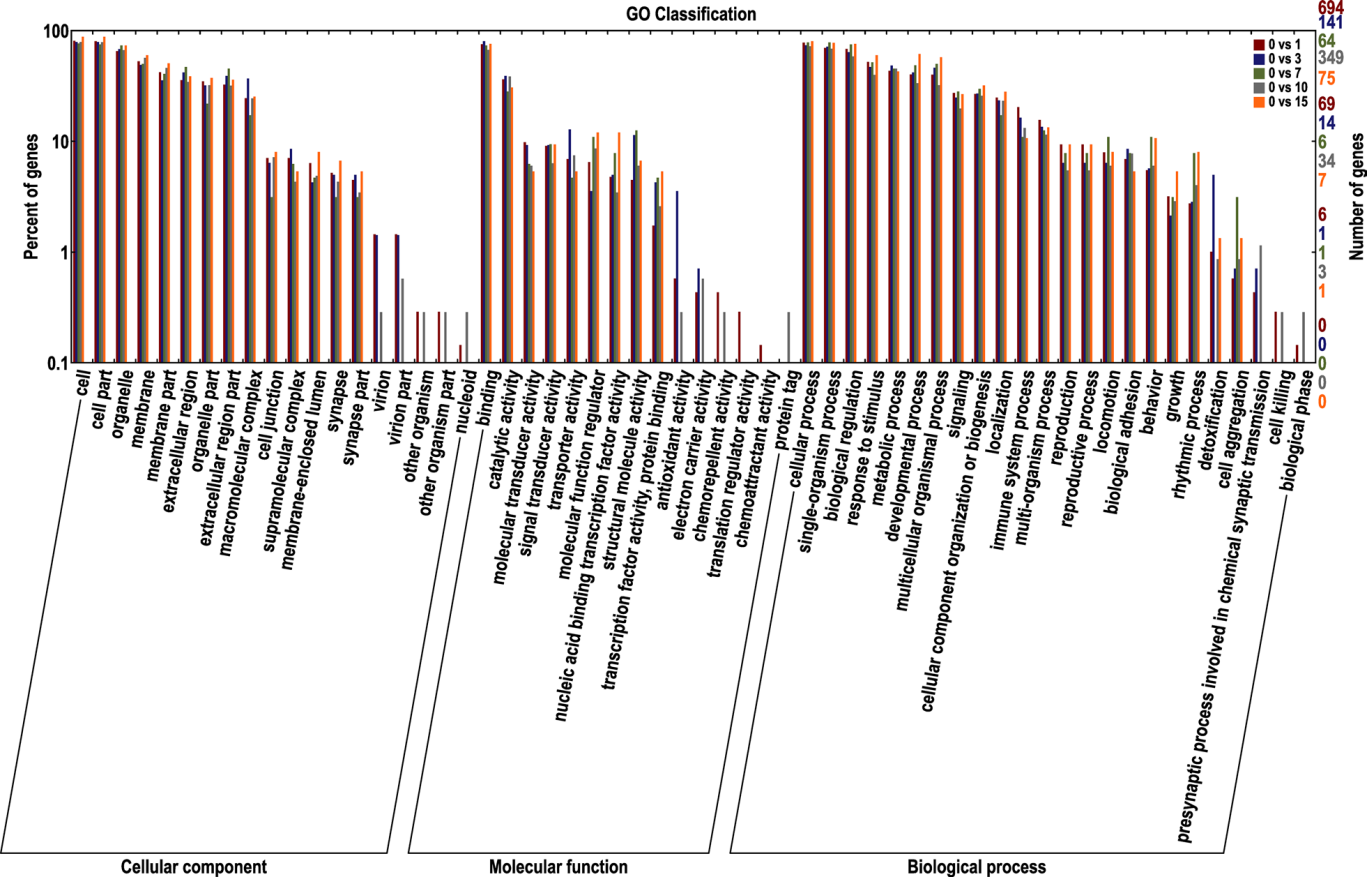
Gene Ontology database (GO) classification of differentially expressed genes (DEGs) at each time point.* Abscissa* GO terms,* left ordinate* percentage of DEGs annotated in GO,* right ordinate* number of DEGs. The figure shows enrichment of secondary GO terms against background DEGs

GO analysis was employed to identify cellular functions and processes that characterize the invasion and survival of *T. spiralis* in murine myocytes. These functions and processes were compared to the lists of DEGs to characterize how the host adapts to parasite invasion. The GO database was used for the functional annotation of DEGs, and the number of genes annotated in the database for each DEG set was 695 (1 dpi), 141 (3 dpi), 64 (7 dpi), 351 (10 dpi) and 75 (15 dpi). Similar to the number of DEGs and DEGs annotated in the KEGG database at each of these time points, the number of DEGs annotated in the GO database decreased significantly at 3 and 7 dpi compared to 1 dpi, suddenly increased sharply at 10 dpi and decreased to a lower level at 15 dpi, which was similar to that observed at 3 and 7 dpi. Genes enriched in secondary GO functions against the background of DEGs are shown in Fig. 6, reflecting the status of all secondary functions, and secondary functions with significant proportion differences indicate different enrichment trends of DEGs. In each of the three main categories (biological process, cellular component and molecular function) of the GO classification, cellular process and single-organism process, cell and cell part and binding and catalytic activity were determined to be dominant.

## Discussion

Unlike previous microarray analyses performed on infected tissue or primary myoblast cultures treated with ML-excretory-secretory products, we conducted a more comprehensive and in-depth host transcriptome study to examine the host response to the muscle stage of *T. spiralis* infection by using advanced RNA-seq technology. In order to maximize the differences in the obtained data, we collected murine diaphragms that had been infected intravenously to enhance differences and to make it easier to discern the host’s pivotal functional genes or pathways. By investigating the host transcriptome at different time points, we are able to understand the changing trends of the host response to *T. spiralis* infection in greater detail. We originally intended to explore host-*Trichinella* interactions in myocytes through daily dual transcriptome dynamic analysis after NBL invasion. However, this aim could not be achieved due to current technical limitations. The percentage of transcriptome data on *Trichinella* after in vivo infection was too low to be useful; therefore, only a time-resolved dynamic profile analysis of host transcription levels at the different time points was performed in this study. In addition, there is some, limited agreement between earlier reported DEGs and our dataset, which probably reflects the differences in how these studies were carried out (e.g., the combination of mouse strain and parasite species, tissue sources, and parasite regulation).

Studies have shown that there is biological variability of gene expression in different individuals, i.e. experimental differences due to subgroups of measurements that are not correlated with the fundamental biology of the system being investigated [15, 16]. To identify DEGs reliably, biological variability should be considered [17]. The most effective and commonly used method for this is to establish biological replicates in the experimental design, i.e. multiple biological samples prepared under the same conditions. Assessment of the relevance of biological replicates is critical to the analysis of transcriptome sequencing data in a project. Prior analyses of high-throughput data, such as those produced using RNA-seq, have demonstrated the need to evaluate and correct biological variability to prevent the misinterpretation of results [18, 19].

The results obtained at each time point showed that there was a trend of a rapid rise-decline-rise again in all the transcripts, DEGs and enriched pathways. These findings may change our present understanding of how organisms react to *Trichinella* infection. The way that organisms resist *T. spiralis*, or the effect of *Trichinella* on organisms, may not be gradual, but erratic. Since NBL were injected into the mice and arrived at the muscles via blood vessels, the physical damage caused by the penetration of the NBL and their secretions would have been extreme stimuli for the muscle cells. Hence, drastic changes in the number of DEGs on the first day after infection are understandable. After this point, *Trichinella* presumably utilizes its own excretory-secretory products to regulate the host’s immune responses, and to make these as stable as possible and suitable for the survival of the parasites. This is thought to explain why there was a subsequent significant decrease in the number of DEGs at 3 and 7 dpi. On 10 dpi, however, there was a massive increase in the number of DEGs, and we tried to examine the reasons for this from the perspective of the growth and development of the larvae. From the time at which NBL penetrate muscle cells to complete nurse cell formation, the only completely developed organ of *T. spiralis* is the stichosome. As the site where most antigenic components are released, the number of stichocytes in the stichosome is greatest from 6 to 10 dpi [20]. Furthermore, in the early stage of infection in muscle (approximately 10 dpi), stichocyte-specific antigens begin to be synthesized and are secreted into the surroundings of the nurse cell for the development of nurse-parasite complexes [21]. This phenomenon may account for the sharp change in the number of DEGs at 10 dpi. Moreover, by performing KEGG enrichment analysis of downregulated genes between 10 and 15 dpi, we could observe that the significantly decreased expression of DEGs from 10 dpi occurred because *T. spiralis* gradually completed its development at this point and stopped absorbing large amounts of energy from the host.

By comparing the differential gene expression libraries obtained from diaphragms at five time points post-NBL invasion, we were able to identify a large number of genes of considerable functional importance with differential expression. Among these genes, only 11 showed consistent tendencies for expression at five time points, suggesting that they may play a pivotal role in host responses to *Trichinella* infection. Some of these genes also appear to be functionally linked. Notably, host genes related to circadian cycles were first noticed in *Trichinella* infection. In addition to genes involved in nutrient metabolism, collagen-related gene and Wnt signaling, which were reported to play a part in regulating the formation of nurse cell, are also in this list [22]. The role of* serpine1* in combating *T. spiralis* infection is also worth considering on account of the fact that serine proteases are one of the two prominent excretory-secretory protein families involved in host-parasite interactions in trichinellosis [23].

*Per2* and* Ciart*, first found in mice by Anafi et al. in 2014, actively participate in the regulation of circadian rhythms and are core regulatory elements of the circadian rhythm in mammals [24, 25]. Various physiological systems in the host that could affect parasite life cycles are under circadian control, such as metabolism and immunity [26, 27]. The effect of immune rhythms on the response to parasitic infections has been elaborated for other parasitic diseases. The infection of mice by *Leishmania* parasites later in the day induced greater injuries than when infection occurred late in the night, and late-day/early night infection induced a more vigorous increase of innate immune cells [28]. In contrast to the immediate inflammatory response in that *Leishmania* study, another study showed that the timing of *Trichuris* infection resulted in a difference in the timing of worm exclusion and antibody induction several weeks later [29]. Furthermore, numerous instances of parasites influencing their hosts’ circadian rhythms have been studied. Human African trypanosomiasis (sleeping sickness) is a parasitic disease that shows the ability of parasites to coordinate host rhythms, as infected patients show confusion in their sleep–wake cycles, which becomes more serious as the disease develops [30]. A thorough analysis of *Trypanosoma brucei*’s capacity to undermine host rhythms has been newly reported [31]. The results of that study showed that the normal circadian rhythms of mice became phase advanced after infection, with eccentric movement happening during the rest phase, which was proposed to be linked to mutations in clock genes only [31]. Despite the fact that there are now many reported instances of the significance of circadian or routine rhythms in host-parasite interactions, only a few studies have reported the impact of *Trichinella* on host circadian clock regulation. The discovery in this study that infection with *Trichinella* caused changes in the expression of host circadian genes may improve our knowledge of the circadian regulation of host-*Trichinella* interactions, and may facilitate the formulation of novel preventive and therapeutic policies for trichinellosis.

When *T. spiralis* NBL penetrate skeletal muscle, they induce the activation and proliferation of satellite cells, which is part of the injury response in adult skeletal muscle. However, these cells do not differentiate into myotubes; instead, they contribute to nurse cell formation [5]. The process of *T. spiralis* nurse cell formation shares many characteristics with that of mammalian myocyte repair, specifically regeneration, proliferation and collagen production [22]. As an NBL-stage-specific serine protease-like protein, *Ts*-NBLsp may function in the larval invasion of skeletal myocytes and nurse cell formation; in support of this, promotive proliferation and upregulation of collagen I and VI expression were observed in our *Ts*-NBLsp transfected myoblasts (unpublished data). NBL may stimulate the production of serpine1 in the host by secreting serine and as a consequence of physical trauma.* Serpine1*, upregulated in some cell models during injury repair, is able to orchestrate wound site stroma and tissue remodeling in the wound microenvironment by restraining plasmin-mediated matrix metalloproteinase (MMP) activation [32]. Additionally, the derepression of plasmin-mediated MMP activation causes degradation of collagen and its decreased deposition, leading to a diminution of fibrotic matrix accumulation in muscles [33]. Recent discoveries emphasize the significance of MMP activities in cell proliferation, migration, and tissue homeostasis [32]. Among the developmental stages of *T. spiralis*, NBL are the most frail but are most exposed to the host immune system. Serpins can help NBL penetrate host defensive barriers more efficiently and avoid immune attack at the same time.

Nurse cells originate from muscle cells during their regeneration. In skeletal muscle, Wnt signaling is involved in myogenesis and muscle regeneration [34]. In a previous study, the* Wnt* signaling-related expression level was detected by using quantitative real-time PCR with PCR arrays in addition to an examination of microarray data sets characterizing the nurse cell transcriptome [9, 22]. Similar to the results of this study, the importance of suppression of Wnt signaling in nurse cells at the plasma membrane level was highlighted by higher upregulation of* Wif1* expression in nurse cells compared with C2C12 myoblasts/myotubes. The genes restraining the canonical Wnt signaling cascade and transmitting the signal of Wnt noncanonical signaling cascades were found to be expressed in nurse cell at high levels, reflecting inhibition of Wnt signaling cascade activity and the predominant character of noncanonical Wnt signaling in the long-term conservation of nurse cell biological functions [22]. Our data further corroborate previous findings that misdifferentiation of infected myofibers and nurse cell functional specificity are regulated by Wnt signaling cascades, according to extrapolation.

In addition to the above, the presence of autoimmune disease-related pathways in downregulated KEGG pathways at 1 dpi attracted our attention. Due to the existence of nurse cells, which occupy an immunologically privileged position, *T. spiralis* and its products can form an immunomodulatory network that may enable the host to cope with miscellaneous hyperimmune-associated diseases [35]. *Trichinella* infection causes Th2-biased immune responses and suppressive/regulatory pathways that diminish excessive inflammation to promote chronic helminth infection in the host and protect host hypersensitivity from autoimmune disorders. Mice infected with *T. spiralis* exhibited fewer arthritis episodes and observably alleviated pathology of collagen-induced arthritis in contrast to uninfected mice [36]. Recent research indicated that *T. spiralis* extracellular vesicles notably attenuated 2,4,6-trinitrobenzene sulfonic acid-induced colitis in mice [37]. Recombinant serine protease from *T. spiralis* NBL has also been proven to have a feasible protective impact against IBD [38]. Moreover, the preventive and therapeutic impacts of *T. spiralis* adult extracts on allergic inflammation in an experimental asthma mouse model and inhibition of autoimmune type 1 diabetes by infection with the gastrointestinal helminth *T. spiralis* have been proven [39]. Notably, unlike the three other diseases that are regulated by Th1 immune responses, asthma, a chronic inflammatory disorder of the respiratory tract, is closely related to abnormal Th2 cell responses [40]. Therefore, the effect of *T. spiralis* on these four diseases cannot merely be generalized by the Th2 response due to *Trichinella* infection. To understand the underlying reasons for this phenomenon, we investigated specific downregulated genes in pathways associated with these four autoimmune diseases and found that the expression levels of class II histocompatibility antigen-related genes (*H2-Aa*,* H2-DMb2*,* H2-DMa*,* H2-Eb1*,* H2-Ab1*, and* H2-DMb1*) were significantly downregulated. Unlike antigenic peptides from extracellular bacteria and viruses that affect antigen-presenting cells, *Trichinella* infection may reduce the activation of Th cells by decreasing the expression of major histocompatibility complex molecules in antigen-presenting cells. Although these four diseases are associated with two different Th responses, *T. spiralis* may play a role in disease by inhibiting Th cell activation and limiting interactions between antigen-presenting and T cells, rather than these subsequently differentiating to each Th phenotype. Understanding the mechanisms and identifying the molecules that regulate the complicated host-*Trichinella* relation could help us to utilize them in the treatment of various autoimmune and allergic diseases. Further studies on DEGs in the KEGG pathway conducted by our research group may help to elucidate the mechanisms governing *Trichinella*-induced immunomodulation of host autoimmunity.

Contrary to assumptions, pathways associated with nutrient metabolism reported in other parasites, such as glycolysis/gluconeogenesis, have not been observed to be affected after NBL invasion. Thus, following the entry of NBL, the diaphragms did not seem to undergo metabolic changes that cause an enhancement in glycolysis or the production of ATP. These findings indicate that NBL probably develop by deriving nutrients in other ways, such as autophagy. Genes related with specific cell biological changes of infected muscle cells at each time point, such as apoptosis, muscle development, myogenesis and regeneration and cell cycle regulation, are also listed in Additional file 5: Table S5. We found that very few well-known genes were differentially expressed, and that only a few DEGs associated with these biological changes were found in the sequencing results of this study. Moreover, numerous disease-specific KEGG pathways were enriched among up- and downregulated genes covered at various time points in this study (i.e., malaria, *salmonella* infection, legionellosis, *staphylococcus aureus* infection, toxoplasmosis, tuberculosis and graft-versus-host disease; see Table 4); this has also been observed in host transcriptional profiling of other parasitic infections [41]. The link between these diseases and our dataset may have other notable implications. However, although it is important to underline that small overlaps between the comprehensive DE profiles in this paper and the genes involved in these pathways, as well as the deviations in scope between the lists, block the validity of contrasts to disease KEGG pathways as presently defined, these results still imply a degree of consistency in the effects of different pathogens on these pathways after host infection.

Although abundant novel discoveries were made in this study, there may be many genes or pathways of significance that were not unearthed, since our study had its limitations. Hopefully, our data can be exploited by other researchers investigating *T. spiralis*, or even other parasites, and may pave the way for further research investigating parasite biology and host-parasite interactions.

## Conclusions

We employed host genome sequence information and RNA-seq technology to systematically analyze information on the transcriptome of the host at different time points after infection. The most extensive and dynamic gene expression responses in host diaphragms were observed during early infection (1 dpi). Only 11 genes were consistently up- or downregulated at all 5 time points, and KEGG pathways enriched at different time points exhibited different characteristics. The results presented here on the *Trichinella*-infected host transcriptome may help to elucidate the regulatory mechanism affecting the growth and development of these parasites and the mechanism underlying the host-parasite interaction.

## Supplementary Information

**Additional file 1: Table S1.** Complete DEG lists for each time point.

**Additional file 2: Table S2.** Eleven genes consistently up- or downregulated.

**Additional file 3: Table S3.** KEGG pathways enriched at 5 time points.

**Additional file 4: Table S4.** Enriched KEGG pathways between 10 and 15 dpi.

**Additional file 5: Table S5.** Genes related to specific cell biological changes of infected muscle cells.

## Data Availability

All relevant data are included in the paper and its supporting information files, except for the data deposited in the Sequence Read Archive database under the BioProject ID PRJNA670288.

## Abbreviations

DEG: Differentially expressed gene
dpi: Days post-infection
GO: Gene Ontology Database
IBD: Inflammatory bowel disease
KEGG: Kyoto Encyclopedia of Genes and Genomes
ML: Muscle larvae
MMP: Matrix metalloproteinase
NBL: Newborn larvae
PCR: Polymerase chain reaction
RNA-seq: RNA sequencing
